# nanoGold and µGold inhibit autoimmune inflammation: a review

**DOI:** 10.1007/s00418-023-02182-9

**Published:** 2023-03-02

**Authors:** Gorm Danscher, Sten Rasmussen

**Affiliations:** 1 Department of Biomedicine, Århus University, Århus, Denmark; 2 Department of Clinical Medicine, Aalborg University, Aalborg, Denmark

**Keywords:** Gold nanoparticles, Gold microparticles, Inflammation, Macrophages, Mast cells

## Abstract

The newest data on metallic gold have placed the noble metal central in the fight for the safe treatment of autoimmune inflammation. There are two different ways to use gold for the treatment of inflammation: gold microparticles > 20 µm and gold nanoparticles. The injection of gold microparticles (µGold) is a purely local therapy. µGold particles stay put where injected, and gold ions released from them are relatively few and taken up by cells within a sphere of only a few millimeters in diameter from their origin particles. The macrophage-induced release of gold ions may continue for years. Injection of gold nanoparticles (nanoGold), on the other hand, is spread throughout the whole body, and the bio-released gold ions, therefore, affect multitudes of cells all over the body, as when using gold-containing drugs such as Myocrisin. Since macrophages and other phagocytotic cells take up and transport nanoGold and remove it after a short period, repeated treatment is necessary. This review describes the details of the cellular mechanisms that lead to the bio-release of gold ions in µGold and nanoGold.

## Introduction

Robert Koch discovered the oligodynamic effect of gold salts and started treatment of rheumatoid arthritis (RA) with gold thiocompounds believing that the cause of RA was bacterial infection (Koch 1890; Richards et al. 2002; Rau 2005). Later it became clear that gold-containing drugs inhibit autoimmune inflammation (Forestier 1932; Freyberg and Block 1941; Fricker 1996). Gold thiocompounds were important for many decades in treating RA and are still used when modern drugs fail (Balfourier et al. 2020a; Castro et al. 2022).

**Fig. 1:**
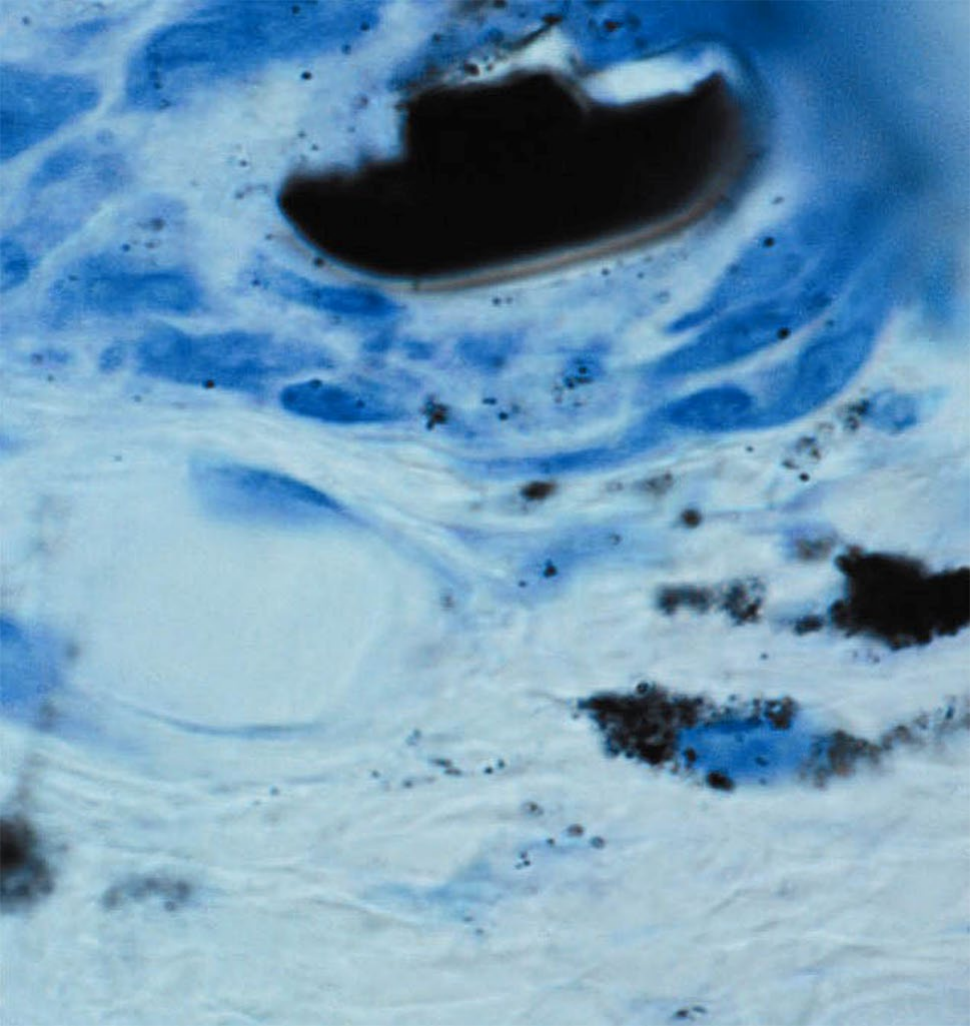
Autometallographic (AMG) silver enhancement of gold ion accumulation in the tissue. The tissue sections are exposed to UV light to reduce the gold ions to metallic gold atoms (Au^0^). The resulting nanosized gold particle is enhanced in an AMG developer to study at LM and EM levels. The AMG-enhanced nanoGold-sized particles accumulate in the lysosomes of macrophages, fibroblasts, mast cells, and secretory granules of the mast cells. In this AMG picture, part of a gold grid is shown in the upper part. The dusty appearance of the tissue close to the gold implant represents gold clusters outside cells. The two loaded cells further away are believed to be macrophages (Danscher 2002)

**Fig. 2:**
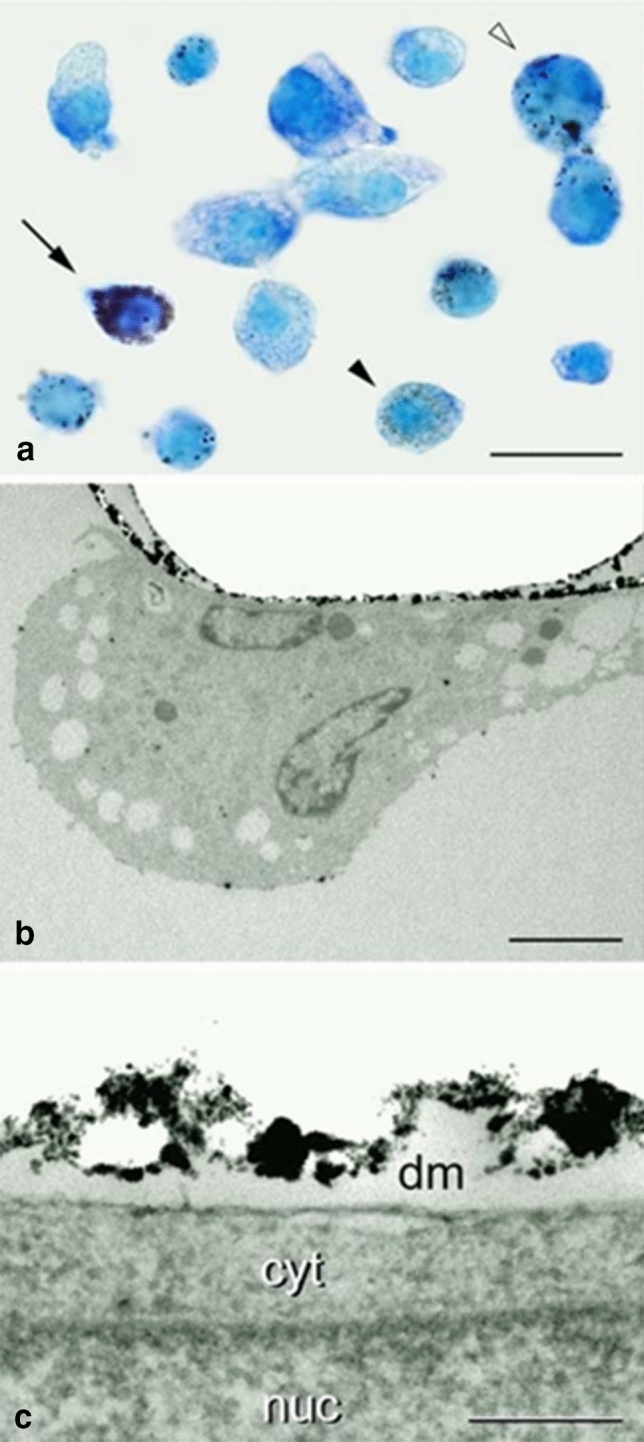
Autometallographic (AMG) illustration of the dissolucytotic release of the gold ions from a gold grid. **a** Light micrograph of AMG silver-enhanced J774 cells, grown on a gold grid, packed with silver-enhanced gold nanoparticles (after UV radiation). (1) Heavily loaded cell (arrow). (2) Cell filled with tiny AMG grains (black arrowhead). Such gold-dusted cells were recorded already after 2 days of exposure. (3) Dissolucytes containing coarse AMG grains after 5 days of exposure (white arrowhead). The picture is a mosaic composed of J774 cells grown on gold surfaces for 2 and 5 days, respectively. Scale bar, 20 µm. **b** Liberation of gold ions into the dissolution membrane. Electron micrograph of a macrophage and its dissolution membrane. The grid to which the cell was attached is removed during the tissue preparation. Notice the AMG-enhanced gold nanoparticles within the membrane. Scale bar, 3 µm. **c** Electron micrograph of the dissolution membrane to show the AMG grains at higher magnification. Dissolution membrane (dm), cytoplasm (cyt), and nucleus (nuc). Scale bar, 200 nm (Larsen et al. 2007)

**Fig. 3:**
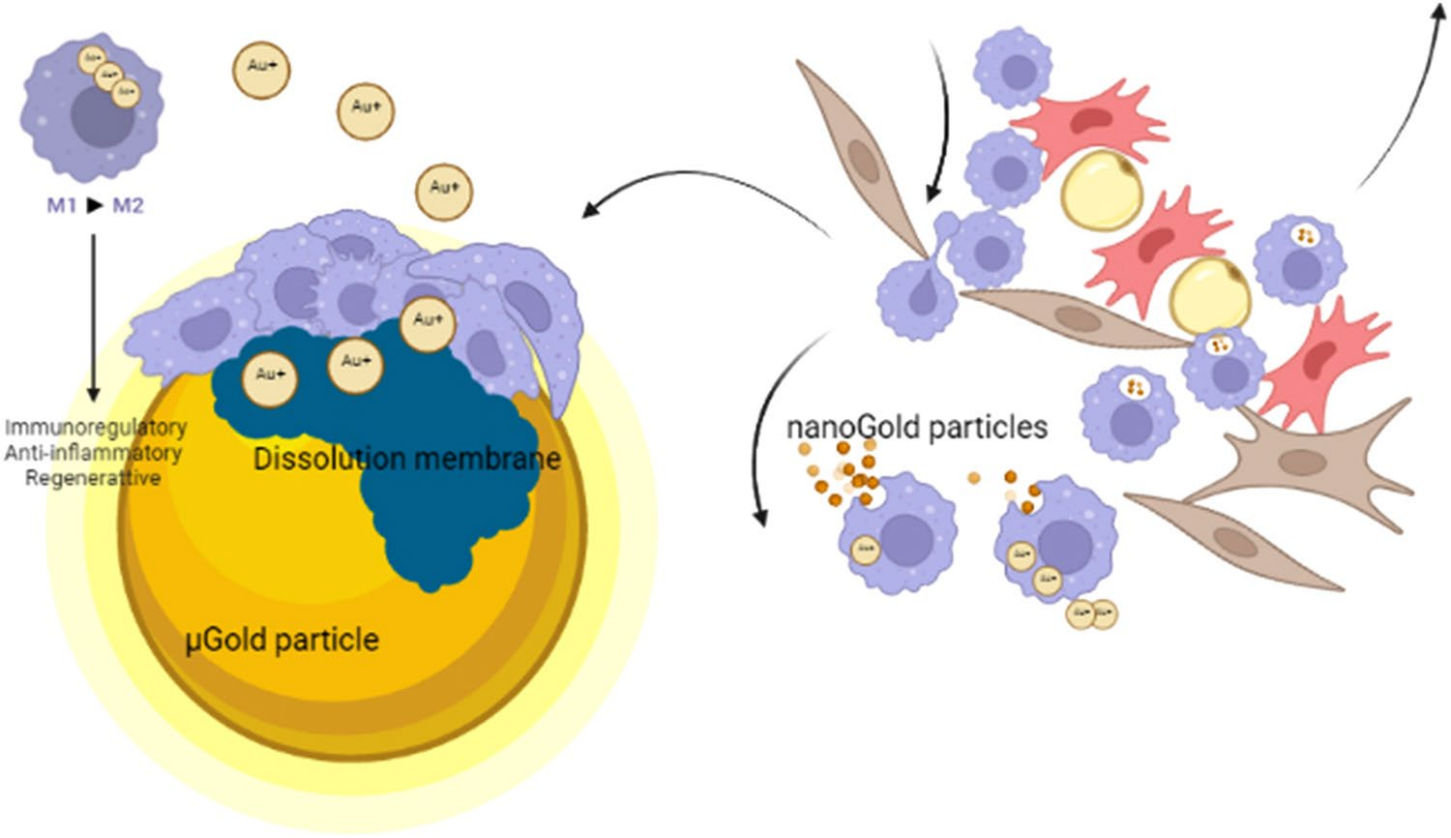
μGold particles (> 20 µm) stay put where they are injected and attract macrophages that liberate gold ions into the intracellular space through dissolucytosis. In contrast, nanoGold particles spread systemically, and in the macrophage lysosomal compartment, there is oxidation to gold ions. The bio-released gold ions (Au^+^), most likely present as dicyanoaurate ions (Au(CN)_2_^–^) that pharmaceutically influence the intracellular microenvironment as well as macrophages, mast cells, and fibroblasts, that is, the bio-released gold ions affect the immune response, inflammation, and regeneration (Rasmussen et al. 2022)

Metallic gold was until recently perceived insoluble in the human body, but research has shown that this is not the case (Danscher 2002) (Fig. 1). Gold ions are proven to be bio-released from metallic gold implants, and it is these dicyanogold ions [Au(CN)_2_^−^] that are responsible for the anti-inflammatory effects of metallic gold (Larsen et al. 2007; Zainali et al. 2010; Danscher and Larsen 2010; Pedersen et al. 2012; Rasmussen et al. 2022) (Figs. 2, 3).

## Historical use in medicine

Several papers prove the immunosuppressing effects of gold by analyzing the outcome of gold-containing drugs on autoimmune diseases (e.g., join inflammation/arthrosis, psoriasis, asthma, psoriasis), impact on cancer, and their antimicrobial (oligodynamic) effects (Chakravarty and Kundu 2019). Gold ions inhibit the cell growth of certain infectious microbes and make these organisms more susceptible to oxidative stress and death (Harbut et al. 2015). Laboratory researchers have demonstrated that even low concentrations of gold nanoparticles exhibit definite inhibitory action against cultures of methicillin-resistant *Staphylococcus aureus* (Kalita et al. 2018).

Gold compounds like aurothiomalate (Myocrisin) are known to reduce inflammation related to rheumatoid arthritis (RA) by releasing gold ions. Treatment with gold thiocompounds did play a significant role in rheumatology for many years. Several clinical trials show that gold ions reduce RA inflammation, reduce radiographic progression, and often induce sustained remission (Williams et al. 1984; Yamashita et al. 1999; Roche 2006).

## Mode of action

In vitro experiments on activated macrophages (Zetterström et al. 2008) showed that aurothiomalate inhibits the extracellular release of high mobility group box chromosomal protein 1 (HMGB1) and has no effect on the secretion of tumor necrosis factor (TNF). They found that aurothiomalate inhibits the endogenous mediators of HMGH1 translocation, interferon beta (IFN-β), and nitrous oxygen (NO). HMBG1 provokes inflammation and is possibly the key process underlying the development of rheumatoid arthritis. HMBG1 is a molecule with dual function. It behaves in one way inside the cell and in another outside (Zetterström et al. 2008; Pisetsky 2017).

Inside the nucleus, HMGB1 plays a role in the transcription of DNA to RNA (Zetterström et al. 2008; Pisetsky 2017). When released from the cell, HMGB1 stimulates the immune system and induces inflammation. There is an unusually high amount of HMGB1 in the synovial tissues and the fluid around inflamed joints. The authors (Zetterström et al. 2008) stimulated mouse and human immune system cells to secrete HMGB1, and then they were treated with aurothiomalate. They found that gold ions blocked the release of HMGB1 from the nucleus reducing the amount available to provoke the body’s immune system and attenuating the inflammatory response. They suggest that gold inhibits the release of HMGB1 by interfering with the activity of two helper molecules, INF and NO, that ease the release of HMGB1 from the cell.

Based on the accumulation of gold in lysosomes of macrophages, fibroblasts, and mast cells (Wojtecka-Lukasik et al. 1986; Shalit and Levi-schaffer 1990; Danscher 2002; Pisetsky 2017), it is tempting to hypothesize that gold ions may have a similar effect on HMGB1 in fibroblasts, mast cells, and other cells of inflammation and may influence the intercellular matrix composition of collagen, reticular, and elastin fibers. Gold-ion-loaded macrophages and mast cells move away over time, and new cells take over (Fig. 3). The µGold particles stay put and continuously expose the matrix and the new cells to gold ions and influence the production of a new intercellular matrix. Contrarily, the macrophages and mast cells will remove the nanoGold from the connective tissue as these cells move away over time (Sadauskas et al. 2007; Balfourier et al. 2020b).

## Aurosomes in macrophages, mast cells, and fibroblasts

A lysosome that contains gold is called an aurosome. The almost explosive increase in our understanding of macrophages (Maruotti et al. 2007), mast cells, and the effect of gold ions on autoimmune inflammation make the introduction of metallic gold implants, e.g., µGold, even more exciting from a treatment point of view (Shapouri-Moghaddam et al. 2018).

Because of its chemical inertness, it was a common belief that metallic gold remains untouched in the body. However, as has been described, gold particles larger than 20 µm, and macro-implants, have been proven to be slowly dissolved by macrophages through a process coined dissolucytosis (Danscher 2002; Larsen et al. 2007, 2008; Seifert et al. 2012) (Fig. 3). Dissolucytosis is a continuous process, dependent only on the number of macrophages, i.e., the inflammatory status, inflammation, or no inflammation of the tissue, and the available gold surfaces at a given location.

The amount of bio-released gold ions is so discrete that all the biologically active Au^+^ or Au (CN)^2−^ (Graham and Dale 1990) remain in a millimeter-sized sphere around the injected µGold particles (Graham 1990). Mast cells, fibroblasts, and other macrophages within this sphere take up the gold ions. The fact that only cells close to the µGold particles become loaded with gold implies that no gold ions are spread to gold-sensitive organs and exclude any toxic effects. The use of µGold, therefore, is considered a safe technique (Danscher 2002; Larsen et al. 2007; Danscher and Larsen 2010).

**Fig. 4:**
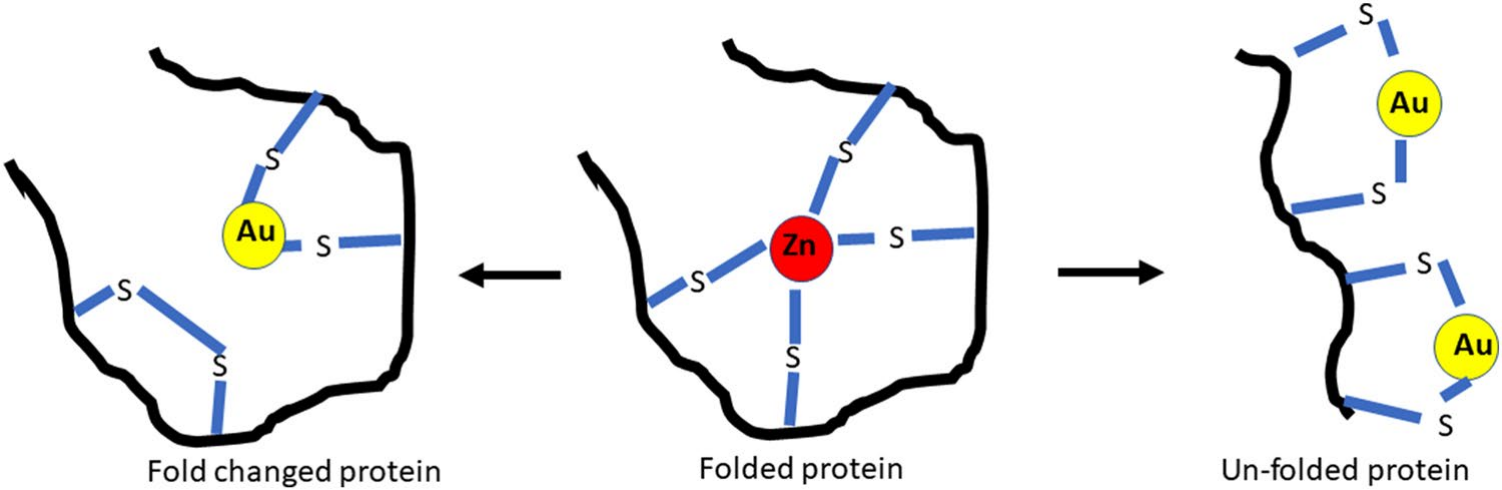
Once in the intercellular fluid and the intracellular compartment, the gold ions act in the same ways as systemically administered gold ions. One of the pharmacological effects may be related to the ability of the gold ions to change the folding of the protein structures. (Rasmussen et al. 2022)

The knowledge about the gold ions while in the intercellular space is sparse. They may present as dicyanoaurate, the gold compound found in the urine of Myocrisin-treated patients (Tepperman et al. 1994; Minakata et al. 2009). The continuous supply of gold ions into the intercellular space by µGold influences the extracellular environment continuously (Figs. 3, 4). Comprehensive studies are required to understand the changes they impose on the extracellular molecules and elements such as collagen, reticular fibers, or elastic fibers. Gold ions may suppress the amount of granulation tissue as the gold ions suppress the production of collagen probably by a reduction in the number of macrophages (Wojtecka-Łukasik et al. 1986; Larsen et al. 2007; Danscher 2017; Balfourier et al. 2020a). It is worth noting that macrophages produce the cytokines PDGF and TGF. They activate fibroblasts producing procollagen, trigger reendothelialization, and stimulate the formation of new blood vessels (Wynn and Barron 2010).

**Fig. 5:**
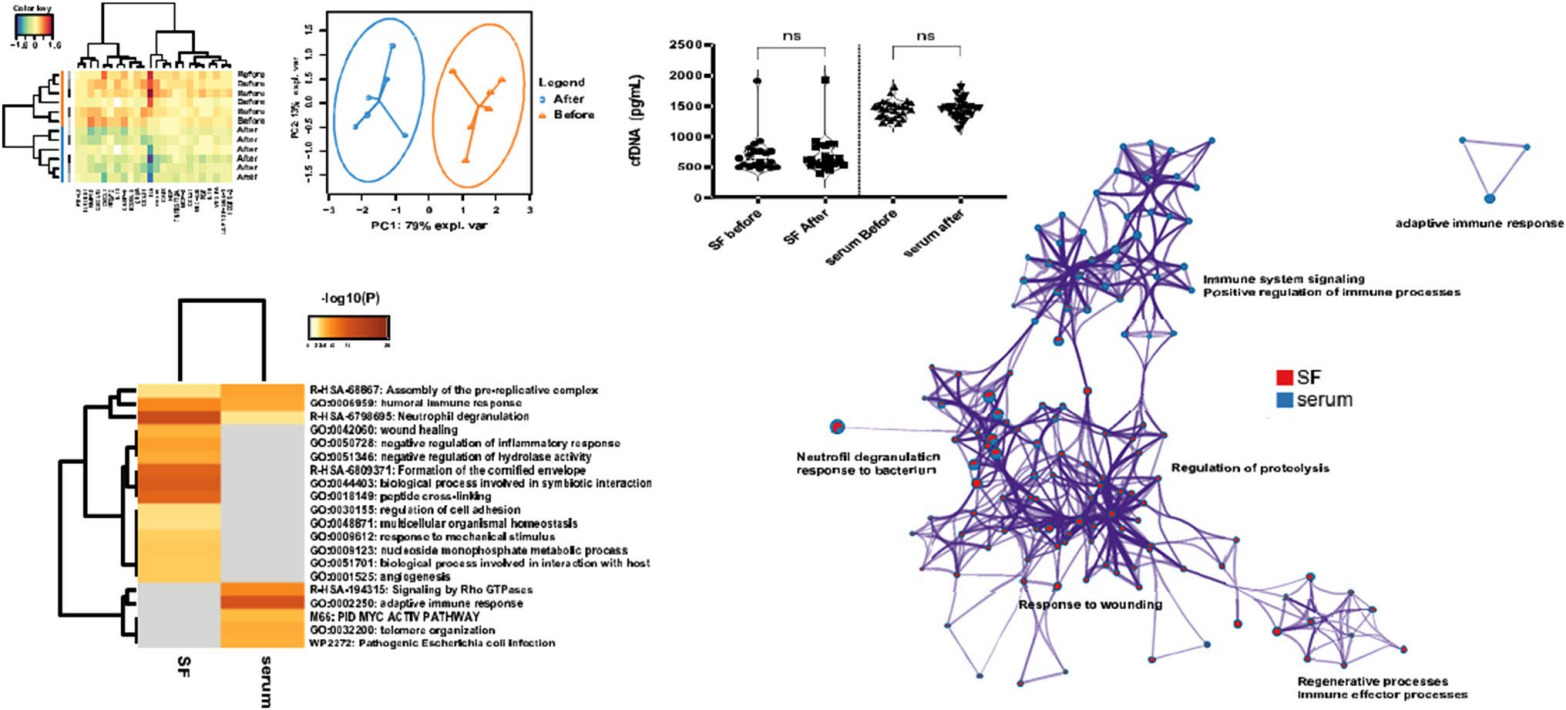
Proteome changes in SF and serum found during treatment indicate involvement of multiple functional pathways and signaling processes, including wound healing, regulation of the humoral and adaptive immune system, and neutrophil degranulation (Rasmussen et al. 2022)

During the inflammatory process, macrophages acquire a plastic phenotype ranging from being pro-inflammatory (M1) to anti-inflammatory (M2) (Fernandes et al. 2020). Different stimuli polarize the macrophages. IFN-g, LPA, and TNF-a polarize to M1, and IL-4, Il-3, Il-10, and TLRs polarize against M2. The M2 macrophage has wound-healing capacities and is pro-chondrogenic. Gold ions initiate an anti-inflammatory response (Seifert et al. 2012), and a clinical pilot study shows this impact of gold ions on the macrophages (Rasmussen et al. 2022) (Figs. 4, 5), though further analysis concerning the polarization is needed.

## nanoGold

With the increasing popularity and use of nanometer-sized gold particles (nanoGold), the impact of the smaller metal particles on inflammation has received considerable attention (Brown et al. 2007; James et al. 2015; Han et al. 2022). When the gold particles are bigger than 20 µm, they are too big for the macrophages to engulf, and the macrophages thus proceed to secrete cyanide compounds and dissolve the gold into cyanide–gold complexes such as Au(CN)^2−^ (Larsen et al. 2008; Zainali et al. 2010; Danscher and Larsen 2010) (Figs. 2, 3). The macrophages degrade nanoGold in a fundamentally different way than µGold. nanoGold particles are oxidized in the lysosomes of the macrophages after being phagocytosed (Carlander et al. 2019) or extracellularly by way of reactive nitrogen species from macrophages (McCarrick et al. 2021). The gold ions diffuse from the lysosomes into the intracellular cytoplasm and nuclei, where they exert their influence on inflammation. nanoGold particles are a possible treatment for rheumatoid arthritis and have a temporal beneficial effect on inflammation (Balfourier et al. 2020a, b).

Lysosomal disintegration of nanoGold causes an intracellular creation of gold ions, having the same effect as gold ions arriving from gold complexes (e.g., Myocrisin) or bio-released gold ions from µGold. While gold complexes and nanoGold spread to all parts of the body and need repeated injections to keep the number of gold ions at a pharmaceutical level (Balfourier et al. 2020b), gold ions released from µGold particles are a purely local process that slowly, but continuously release local pharmaceutical levels of active gold ions into the intercellular space (Rasmussen et al. 2022).

For more than 50 years, gold-containing drugs like Myocrisin have been in use for the treatment of different autoimmune maladies, and an overwhelming amount of literature has been gathered proving that gold ions are potent immune suppressors (Petersen and Bendtzen 1983). Dicyanogold ions (Au(CN)_2_^–^) affect lymphokine production and are a common metabolite found in blood and urine samples from patients treated with different gold-based drugs (Tepperman et al. 1994, 1999).

## µGold

As the release of gold ions from gold microparticles is carried out by macrophages that settle on the gold surface, the surface area per weight of gold must be as high as possible. The microparticles must not be smaller than particles ≥ 20 µm, or they will be phagocytosed by the macrophages and treated in the lysosomes like gold nanoparticles. When placed into the tissue, proteins are adsorbed to the surface, creating a nanometer-thick membrane (Danscher 2002; Seifert et al. 2012).

The macrophages organize the initial molecular “membrane” between themselves and the gold surface, creating a dissolution membrane (Danscher 2002; Larsen et al. 2007) (Figs. 1, 2). The macrophages control the chemical conditions and milieu in the dissolution membrane and secrete, among others, cyanide ions into the membrane. At the surface of the gold microparticle and the optimal oxygen tension and pH, the macrophages oxidate gold atoms to monovalent gold ions (Au^+^). The gold ions then extracellularly in the dissolution membrane bind to cyanide ions (CN)^−^ and create dicyanoaurate ions [Au(CN)^2−^]. The dicyanoaurate ions diffuse from the dissolution membrane into the intercellular space, bind to soluble and membrane-bound proteins containing sulfur, and are taken up and concentrated in the lysosomes of the cells (Larsen et al. 2007; Minakata et al. 2009; Danscher and Larsen 2010). Some of the gold-loaded proteins, positioned in the cellular membrane of the dissolucytotic macrophages, themselves, in local mast cells, fibroblasts, and the interstitial fluid, are found in the lysosomes and mast cells, additionally in the basophilic granules (Danscher 2002; Danscher and Larsen 2010) Using the histochemical technique autometallography (AMG) (Jacobsen et al. 1989; Danscher 2002; Danscher and Stoltenberg 2006), gold ions liberated from gold implants can be traced in macrophages, mast cells, and fibroblasts and fibers, the same cells that take up gold ions in patients injected with aurothiomalate (Myocrisin) (Møller-Madsen et al. 1987; Graudal and Møller-Madsen 1988; Jacobsen et al. 1989).

If the tissue is in a state of inflammation when injecting the gold microparticles, or if the inflamed tissues already contain gold microparticles, there may be a higher release of gold ions compared with tissues not affected by inflammation. The reason for this effect is not yet determined but is likely in part related to the significant increase in the number of macrophages associated with inflammation. This may explain the clinical findings in patients with knee osteoarthritis and effusion (Rasmussen et al. 2022).

Another important observation is that metal implants in animal studies stay well fixed when there is a partial coating with gold (Zainali et al. 2013) but exhibit inferior fixation when there is a complete gold coating (Zainali et al. 2009). A third study using particulate gold in bone allograft influenced the cells adjacent to the gold microparticles without reducing the implant fixation (Zainali et al. 2010). These studies indicate that gold ions reduce inflammation and the number of granular cells, fibroblasts, and collagen (Danscher and Larsen 2010) and may reduce the capsular membrane around the implants. Adding gold to the surface of an implant may cause a substantial prolongation of the stability of the bone-metal zone (Zainali et al. 2010, 2013). This observation calls for further evaluation.

An important issue when applying metallic gold is whether magnetic resonance imaging (MRI) may cause a negative effect. One study investigated the matter using upper eyelid gold weight implants placed in an open chamber exposed to 1.5 T and subcutaneously in six rats (Marra et al. 1995). There were no displacements or adverse effects, suggesting that it is safe to perform MRI on patients treated with µGold particles.

## nanoGold versus µGold

There is a temporary and beneficial effect of nanoGold on autoimmune inflammation (Balfourier et al. 2020b). In contrast, µGold induce a long-lasting up to 2-year suppression or even a shutdown effect on the inflammation in joints suffering from osteoarthritis (Rasmussen et al. 2022) (Fig. 5). nanoGold may be less acutely toxic than gold-containing drugs such as Myocrisin. However, there are no studies of the long-termed effects of gold nanoparticle treatment on humans, but one study on acute and chronic administration of nanoGold finds they cause DNA damage in the cerebral cortex of adult rats (Cardoso et al. 2014). It is general knowledge that nanoGold will enter an unknown multitude of cells, including neurons and tubular cells of the kidney, that might make long-term treatment problematic. Moreover, the liver is known to be central in the accumulation of nanoGold (Sadauskas et al. 2007, 2009).

## Increased release of gold ions in inflammatory tissue

If the tissue containing µGold is or becomes inflammatory, the amount of released gold ions increases, most likely because the amount of dissolucytotic macrophages increases until the inflammation has ceased (Danscher et al. 2008 unpublished results). In a transplantation study of the ability of gold microparticles to suppress inflammation and delay rejection of the transplanted tissue, gold microparticles cause a pronounced decrease in the formation of granulation tissue (Danscher et al. 2006). As both macrophages and mast cells are major players in the inflammatory process (Tamang et al. 2012; Xu and Shi 2012), placement of pure metallic gold (99.99%) in inflammatory tissues will interact profoundly with the inflammatory processes, leading to repression or even to a total cessation of the inflammation (Rasmussen et al. 2022) (Fig. 5).

More speculative is the notion that the gold ions cause the formation of new chondrocytes in the gold-treated joints, but the finding that gold particles injected into brain lesions evoke neogenesis of glial cells supports the possibility (Pedersen et al. 2012). Whether osteophytes may be affected by treating a diseased joint with µGold or may stay unaffected and not increase in size is not known.

As mentioned above, gold nanoparticles are taken up by macrophages and concentrated in the lysosomes (Sadauskas et al. 2007). In the lysosomes of the macrophages, oxidation turns nanoparticles into monovalent gold ions (Au^+^) (Carlander et al. 2019). The lysosomes end up having a load of gold ions as after the extracellular dissolucytosis of µGold or after injection of Myocrisin. Electron microscopy detects the presence of gold ions in the lysosomes (Danscher 2002; Carlander et al. 2019) (Figs. 1, 2).

## Conclusion

Pain or nociception is a protective sensation that, in physiological conditions, is of paramount importance to humans and other living organisms to the extent that it must be considered essential for survival.

Thus, finding an effective treatment that suppresses inflammation and reduces pain is a topic for basic researchers and clinical doctors. A safe local gold cure might comprehend such qualities as the goal of the ongoing research on bio-released gold ions from gold implants.

From experimental and empirical evidence, and soon hopefully supported by more clinical studies, we know that the number of gold ions released from metallic gold implants is sufficient to cause a local effect around the implant (Seifert et al. 2012; Pedersen et al. 2012; Rasmussen et al. 2022) (Fig. 5).

We do now know whether the pain-reducing effect of gold ions results from anti-inflammatory characteristics or includes a direct impact of gold ions on pain perception. However, the visible decline in the local edema suggests that inhibition of histamine release from mast cells by monovalent gold ions might play a role (Shalit and Levi-Schaffer 1990). Since the scientific proof of macrophage-caused liberation of gold ions in connective tissue and the brain is established (Danscher 2002), several animal models of such maladies (Lie et al. 2011; Jæger et al. 2012; Pedersen et al. 2012; Märki et al. 2018) and a human trial (Rasmussen et al. 2022) prove the rational use of gold as treatment of noninfectious or autoimmune inflammation.

## Data Availability

The references and data analysed during the current study are available from the corresponding author on reasonable request.
